# Quantifying the direct cost benefits of vestibular telemetry using the CAVA system to diagnose the causes of dizziness

**DOI:** 10.1186/s12962-022-00413-9

**Published:** 2023-01-13

**Authors:** John S Phillips, Jacob Newman

**Affiliations:** 1 Department of Otolaryngology, Norfolk & Norwich University Hospitals NHS Foundation Trust, Colney Lane, Norfolk NR4 7UY Norwich, UK; 2 University of East Anglia, Norwich, UK

**Keywords:** Dizziness, Vertigo, Diagnosis, Health economics

## Abstract

**Background:**

It can be challenging to diagnose the cause of a patient’s dizziness. Patients face significant delays before receiving a correct diagnosis as they will undergo many diagnostic tests under several different medical specialities. As well as prolonging the suffering of patients, these problems place a significant financial burden on health services worldwide. We have developed a wearable medical device which has the potential to diagnose the cause of a patient’s dizziness using vestibular telemetry captured over a thirty-day period. We sought to quantify the potential direct cost savings of an alternative diagnostic pathway using our diagnostic device.

**Methods:**

In this work, we identified the existing diagnostic pathways followed by patients reporting dizziness to their General Practitioner, and modelled the best and worst-case direct costs of providing a patient with a correct diagnosis. We estimated the potential cost of our alternative pathway, and calculated the cost savings this could provide to the NHS.

**Results:**

The results show that our alternative diagnostic pathway could reduce the time and direct cost associated with providing a correct diagnosis. We present a potential indicative cost-saving of between £631 and £1305, per patient.

**Conclusion:**

Our alternative diagnostic pathway would reduce the time taken to correctly diagnose patients with vertigo. This in turn would facilitate faster access to targeted treatments, reduce unnecessary interventions, and reduce the suffering of patients. These improvements would also lead to other savings, such as reducing the amount of sick leave taken by patients to attend appointments, and freeing up of NHS time to see other patients.

## Background

Dizziness is an extremely common symptom, with nearly 25% of the population reporting ‘significant’ dizziness at any given time [1]. Dizziness can be caused as a result of malfunctions in many different organ systems affecting the inner-ear, the brain and the circulation. When patients report “dizziness” to their clinician, they may be referring to symptoms ranging from light-headedness to violent and prolonged attacks of the world spinning around them [2]. It can be challenging to diagnose the cause of a patient’s dizziness, leading to multiple hospital visits and long delays before patients receive a correct diagnosis and appropriate treatment [3]. Dizziness is therefore a huge burden on health services [4]. To overcome these limitations of the conventional diagnostic pathways for patients reporting dizziness, we have developed the Continuous Ambulatory Vestibular Assessment (CAVA) system.

The CAVA system includes a medical device which continuously records eye- and head-movements over a period of thirty days. The vestibular telemetry provided by the CAVA system provides vital information to aid a clinician’s assessment of patients reporting symptoms of vertigo. Having recently evaluated this system in healthy volunteers [5, 6], and presently in patients suffering from vertigo [7, 8], we now wish to determine the potential direct cost benefits of deploying the CAVA system into routine medical care.

In this article, we determine the common diagnostic pathways currently employed to diagnose patients with dizziness. We explore the medical specialities that patients are commonly referred to, the subsequent tests they undergo, and the direct costs associated with these activities. We present an estimated range of indicative costs incurred by the NHS in diagnosing a typical patient, compare these figures to an estimate of an alternative pathway using the CAVA system, and present the potential cost-savings.

## Methods

As there is no single source detailing the diagnostic pathways followed by patients reporting dizziness, we have compiled information from a number of freely available sources. Our methods for defining and quantifying the direct costs associated with existing diagnostic pathways were broadly divided into the following areas: (a) describes how we identified the current diagnostic pathways, including the number of appointments and the medical specialities involved. (b) lists the tests commonly undertaken, their associated costs and the sources of this information. (c) provides our methodology that modelled the best- and worst-case patient pathways. (d) justifies the estimated costs of our proposed alternative pathway.

### The current pathways

A study by the US National Institutes of Health reported that patients will see 4.5 clinicians on average before receiving a diagnosis. Reflecting the way that patients will be referred to multiple specialities, in our work we assumed that patients would attend 2 new appointments and 2.5 follow-up appointments on average.

Patients with dizziness are predominantly referred to three distinct medical specialities: Ear, Nose and Throat (ENT), Cardiology and Neurology. Patients may also be referred to General medicine, Geriatric medicine and to falls clinics. To simplify our analysis and to reflect the common diagnostic pathways, we have combined these additional specialties into a “Medical Specialities” subheading. The costs of new and follow-up appointments for each speciality were provided by the Norfolk and Norwich University Hospitals NHS Foundation Trust. For the Medical Specialities subheading, new and follow-up appointment costs were averaged across specialities.

### The diagnostic tests

We asked clinicians from ENT (and Audiology), Cardiology, Neurology and Older People’s Medicine to provide a list of the tests they would request for patients reporting symptoms of dizziness. The costs of these tests were obtained from sources including the 2019/2020 NHS tariff [9], the National Institute for Health and Care Excellence (NICE) [10–12], and from the British Medical Journal (BMJ) [13]. All costs are in GBP, and we present all costs to the nearest Pound. The full details of how we analysed these costs and the sources used are shown in Table 1.

**Table 1:** The details and sources of data for the costs of diagnostic tests used to assess patients reporting dizziness

Diagnostic test	Source	Notes
CT Scan	2019/2020 NHS Tariff (9)	Average across all types of CT scan.
MRI Scan	2019/2020 NHS Tariff (9)	Average across all types of MRI scan.
ECG	2019/2020 NHS Tariff (9)	Cost of electrocardiogram monitoring or stress testing, for congenital heart disease.
24-hour ECG	2019/2020 NHS Tariff (9)	Cost of electrocardiogram monitoring or stress testing.
Audiometry	2019/2020 NHS Tariff (9)	Aged 19 and over.
Balance assessment	2019/2020 NHS Tariff (9)	-
24-hour BP	2013 estimate from NICE (10)	Unit cost.
TFTs	2019 estimate from NICE (12)	Median cost of TSH + FT4.
FBC	2015 estimate from NICE (11)	-
Bone profile	2014 estimate from BMJ (13)	-
U & E	2014 estimate from BMJ (13)	-

### The range of costs

In order to provide a fair representation of the direct costs associated with the current pathways, we calculated a range of costs to reflect a likely best and worst-case scenario. We expect that the true average cost will lie between these values. The total cost of a patient’s diagnostic journey is determined by the cost of all appointments attended plus the cost of all tests performed. For the best-case scenario, we have assumed that over the course of seeing two separate specialists, patients are likely to undergo at least one type of scan. In the worst-case, patients may undergo every test in our list. These scenarios represent the likely average cases rather than those of outlier patients who might require duplicated tests or no tests at all. The best and worst-case costs are calculated as follows:

#### Best case

2 new appointments + 2.5 follow-up appointments + 1 CT scan.

#### Worst case

2 new appointments + 2.5 follow-up appointments + 1 of every test.

### The CAVA system pathway

The CAVA system has shown to be capable of identifying periods of pathological nystagmus and can detect discriminating features for three of the most common inner-ear causes of dizziness [7, 8]. Different patterns of nystagmus have also been shown to be unique for central and cardiac causes of vertigo [14, 15]. The effectiveness of the CAVA system has been detailed in other publications [5–8]; further diagnostic accuracy trials will commence throughout the United Kingdom in 2023. In parallel with this work, detailed data will be collected to consider real life data to quantify a broad range of variables, including indirect health system costs, indirect patient costs, implications for quality of life and time efficiency. It is likely that the CAVA system will be able to automatically diagnose a range of conditions resulting in dizziness. Our analysis assumes that the CAVA system will be able to provide 100% diagnostic accuracy at discriminating the causes of a patient’s dizziness. As such, an alternative pathway incorporating the CAVA system would not require visits to other medical specialities or for other tests to be carried out.

We expect that the CAVA system would be given to patients in primary care, and so we have not included new or follow-up appointment costs for this pathway. The total cost of this pathway is based on the material and tooling costs for 35 CAVA devices, and assumes each device has a lifespan of 50 patients. This figure is likely to be an overestimate, given that mass production of the device would dramatically reduce the per unit costs and there is no reason to suspect that the devices will have a finite lifespan. The cost we have estimated for this pathway also includes consumable accessories, such as batteries and single-use ECG electrodes.

## Results

**Fig. 1:**
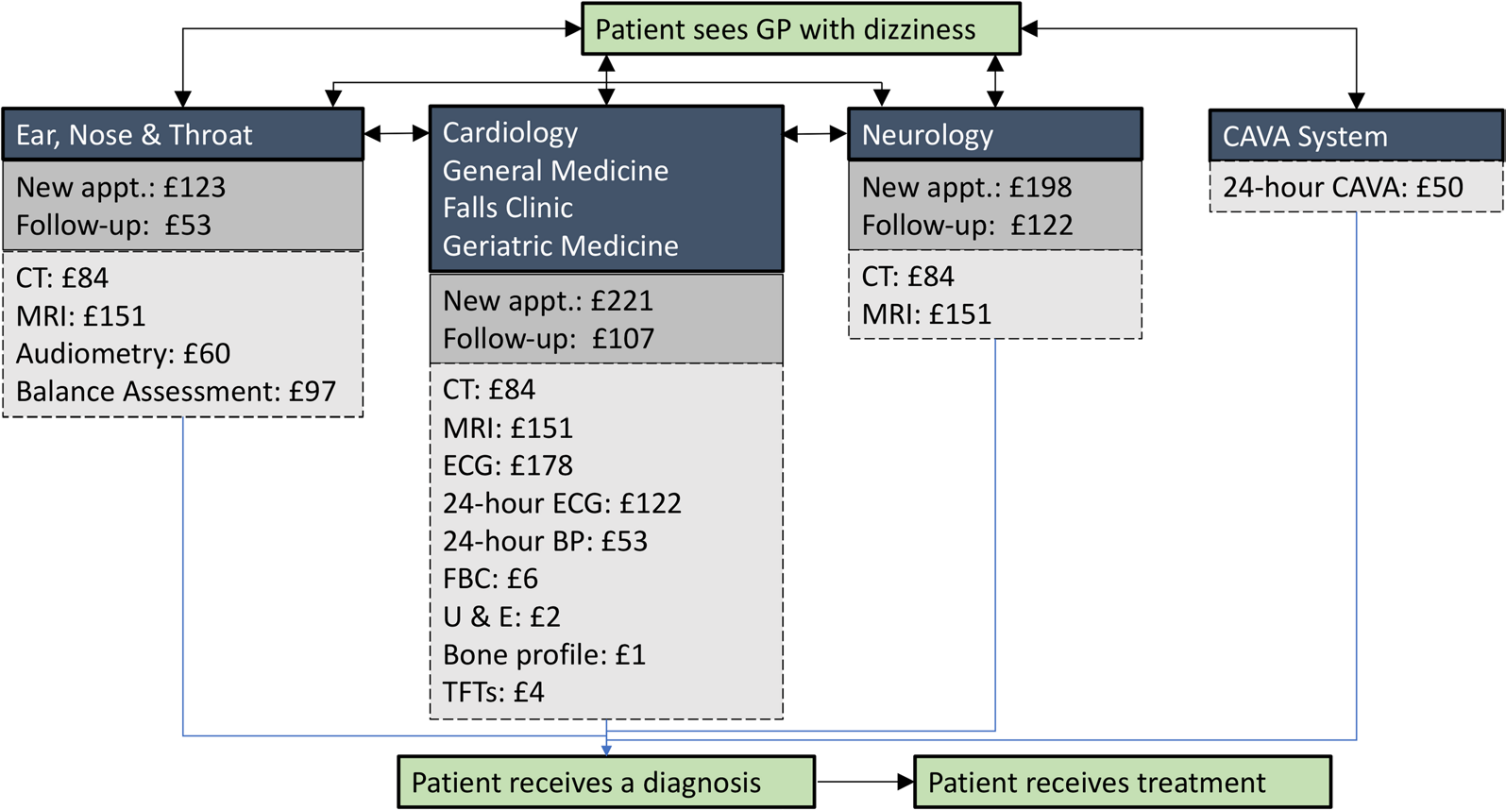
A diagram showing the current diagnostic pathways and the associated costs for patients reporting dizziness to their GP. An alternative pathway consisting of the CAVA system is also shown

Figure 1 is a diagrammatic representation of both the existing pathways for diagnosing patients reporting dizziness, and a new pathway incorporating the CAVA system. Currently, patients will commonly be referred to three separate medical specialities, including ENT, Cardiology and Neurology. To a lesser extent, they may also be referred to general medicine, geriatric medicine and falls clinics. The specialities in Fig. 1 are linked by bi-directional arrows, representing how patients will likely be referred to and from different specialities over the course of their diagnostic journey. By contrast, we expect that the CAVA system could be employed as a separate test, which by itself would provide the cause of a patient’s dizziness.

For the existing pathways, we estimated the best case to cost approximately £681, rising to £1355 in the worst case. These costs include a minimum of 1 diagnostic test and a maximum of 11 unique tests. Comparable to the cost of 24-hour blood pressure monitoring, we estimate the CAVA system pathway would cost £50, per patient. Therefore, the potential savings associated with an alternative pathway using the CAVA system lies between £631 and £1305.

## Discussion

In this article we have identified the existing diagnostic pathways that are commonly taken by patients reporting dizziness to their GP and presented an alternative pathway involving the CAVA system. Implementation of this alternative pathway would lead to a number of benefits for patients and healthcare providers. For example, it would reduce the time to diagnosis, reducing the suffering of patients, and the anxiety associated with attending multiple hospital appointments and undergoing repeated medical tests. There are also financial and employment implications for patients taking large amounts of time off work due to ill health or to attend hospital appointments. Freeing up valuable appointment times would provide NHS services with the flexibility to treat patients with other conditions too, leading to improved patient outcomes and further economic savings in these areas. A limitation of this current work, is that it has only considered direct costs, rather than the broader range of direct and indirect costs incurred when individuals present with dizziness. Nevertheless, this initial piece of work will support future work which has been funded to provide a more comprehensive assessment of costs, efficacy, efficiency, and quality of life.

In researching the data to include in this analysis, we have found that there is much ambiguity and variation in the way that costs are reported by different NHS trusts. For example, we have located freedom of information requests showing that several trusts referenced different entries in the NHS tariff when questioned about the same diagnostic test. In the interest of clarity and accuracy, there is a need for NHS institutions to report costs consistently to allow fair comparisons to be made. This is especially relevant today, as the COVID-19 pandemic has had a dramatic and lasting impact on world economies, which has motivated the need to find financial savings wherever possible.

The results presented here were derived from a range of sources rather than from empirical patient data. In future, we intend to carry out a comprehensive study into the potential economic and patient benefits of the CAVA system, using actual patient data gathered during a large clinical investigation. This study will seek to evaluate the CAVA system’s capability to diagnose patients with three of the most common inner-ear causes of vertigo. We will examine the diagnostic pathways followed by real patients and will determine the actual costs incurred by those patients. We are keen to compare the indicative costs described here with the results of that analysis. Our ultimate goal is to ensure that all potential benefits of the CAVA system are passed onto both health services and patients.

## Conclusions

We have found that patients will visit many specialists and will undergo many diagnostic tests in pursuit of a correct diagnosis. Our alternative pathway is expected to render many of these visits and tests redundant, by quickly and accurately determining a patient’s likely diagnosis, facilitating targeted access to treatment and specialist care. We estimate the potential savings of an alternative treatment pathway using the CAVA system will fall between £631 and £1305, per patient. In addition, patients and healthcare providers will benefit by reducing the number of unnecessary interventions given to patients. Although we have made a number of assumptions in our modelling, both under- and over-estimating the associated costs, we expect that the true cost-savings are likely to be much higher than indicated. For example, our analysis does not consider the significant impact on health services of patients attending emergency departments due to dizziness, which was estimated to be in excess of US$4 Billion in 2013 [4].

## Data Availability

The datasets used and/or analysed during the current study are available from the corresponding author on reasonable request.
